# Prevalence of Metabolic Syndrome Among Adults Aged 50 and Above and Associated Factors: A Cross‐Sectional Study From Ardakan Cohort Study on Aging (ACSA)

**DOI:** 10.1002/hsr2.70508

**Published:** 2025-03-04

**Authors:** Elham Hooshmand, Isa Akbarzade, Delaram Delbari, Mahtab Niroomand, Fatemeh Ghavidel, Mohammad Saatchi

**Affiliations:** ^1^ Iranian Research Center on Aging University of Social Welfare and Rehabilitation Sciences Tehran Iran; ^2^ Department of Internal Medicine, School of Medicine Shahid Beheshti University of Medical Sciences Tehran Iran; ^3^ Department of Biostatistics and Epidemiology University of Social Welfare and Rehabilitation Sciences Tehran Iran

**Keywords:** abdominal obesity, aging, insulin resistance, metabolic syndrome, pre‐diabetes

## Abstract

**Background and Aim:**

Metabolic syndrome (MetS) is one of the most important late‐life diseases. This study was conducted to estimate the prevalence of MetS in the elderly population (over 50 years old) of Ardakan, Iran.

**Methods:**

This cross‐sectional study was from the first phase of the Ardakan Cohort Study on Aging (ACSA). The MetS was defined based on the Adult Treatment Panel (ATP III) definition. All anthropometric data from ACSA were measured and recorded by trained staff. Standard laboratory methods were used to conduct all blood tests. Physical activity was scored based on the Physical Activity Scale for the Elderly (PASE), and the quality of sleep was assessed by Pittsburgh Sleep Quality (PSQI). Multivariable logistic regression was used to assess associated factors with MetS (α > 0.05).

**Results:**

A total of 5944 older adult participants were entered into this study analysis. The mean age was 62.5 ± 8.0, and 50.5% were female. The prevalence of MetS was 66.81% (95% CI: 65.59–68.00) in this study. Based on the multivariable regression and adjusted odds ratio, six variables of age (OR: 1.05), BMI (OR: 1.19), family history of diabetes (OR: 1.48), hypertension (OR: 1.31), physical activity score (OR: 0.99), and having a fatty liver (OR: 1.71) were associated with having MetS.

**Conclusion:**

The results of this study showed that the prevalence of metabolic syndrome was high in the older population of Iran. Also, the most prevalent MetS component was triglycerides, and HDL was the least prevalent. Hence, based on these findings, tailored interventions seem necessary to control this syndrome in older Iranian populations.

## 1 Introduction

In developed and underdeveloped countries, noncommunicable diseases (NCD) have become the most prominent cause of morbidity and mortality due to the successful eradication of many old infectious diseases. Metabolic syndrome (MetS), syndrome X, insulin resistance, or deadly quartet, has been the major global health problem among these NCDs [1]. The MetS comprises several known cardiovascular risk factors, including insulin resistance, obesity, atherogenic dyslipidemia, and hypertension. Several mediators, mechanisms, and pathways are involved in each of these conditions, and they are interrelated [2].

The prevalence of MetS is estimated to be between 20% and 25% in adults around the world; in developing countries, the trend is most noticeable in the urban population [3]. It is estimated that 30% of Iranian adults (over 24 million individuals) have metabolic syndrome [4]. Those with metabolic syndrome have twofold odds of dying, threefold greater odds of having a heart attack or stroke, and fivefold greater odds of developing type 2 diabetes [3, 5]. Also, this syndrome increases the incidence of liver, colorectal, and bladder cancers [6]. Also, in the Middle East, the attributable risk of MetS for cardiovascular disease, coronary heart disease, and stroke are estimated to be 15.87, 11.7, and 16.23, respectively [7].

Among Iranian adults, the prevalence of MetS increases significantly with age, ranging from 12.1% in the 20–29 age group to 51.7% in the over‐60 age group [4], and based on this, age plays a vital role in occurrence and outcomes of this disease [8]. Proper management and prevention of MetS are necessary for adopting several national programs, including lifestyle modification, medical interventions, and public education about NCD risk factors [9].

Clinical manifestations of the MetS are more likely to occur in genetically susceptible individuals. However, acquired risk factors such as obesity, physical inactivity, and a high‐fat diet are also major contributors [10]. The three main aspects that cause the occurrence of MetS, a cluster of risk factors for cardiovascular diseases and many other diseases, include genetics, nutrition, and physical activity. It is possible to prevent or even prevent the development of this syndrome by controlling and checking factors related to this triangle of factors [11, 12].

The prevalence of MetS could be different in various age groups and geographical areas. This syndrome comes with aging, and the elderly population is prone to have higher rates of MetS [7, 13]. Since this syndrome usually comes before type 2 diabetes [14], and Yazd province in Iran is known to have a high prevalence of diabetes [15], this area and this age group are perfect for this study. This study was conducted to estimate the prevalence of MetS in the elderly population (over 50 years old) of Ardakan, Iran.

## 2 Methods and Materials

### 2.1 Study Design and Population

This study was conducted as part of the Ardakan Cohort Study on Aging's (ACSA) cross‐sectional phase. This cohort study, which began in 2020, focuses on over 50 adults in a central part of Iran named Ardakan, Yazd. The study's further details were published recently [16]. This cohort study complies with the Helsinki Ethical Principles For Medical Research Involving Human Subjects [17]. The sample size was based on the all‐in census method. The informed consent form was signed by all participants of this study prior entering to the study. This study has received ethical approval from the Ethics Committee of the Deputy of Research at the University of Social Welfare and Rehabilitation Sciences (IR.USWR. REC.1394.490).

### 2.2 Metabolic Syndrome Definition

Participants' general and baseline information was collected through ACSA checklists. MetS was defined using the criteria below, according to which the presence of any three of the following five items indicates the presence of MetS [1]:

1. Abdominal obesity: waist circumference ≥ 95 cm.
2. Serum triglycerides ≥ 150 mg/dL (1.7 mmol/L) or specific treatment or medications for this lipid abnormality.
3. Serum high‐density lipoprotein (HDL) cholesterol < 40 mg/dL (1 mmol/L) in men and < 50 mg/dL (1.3 mmol/L) in women or specific treatment or medications for this lipid abnormality.
4. Blood pressure ≥ 85/130 mmHg treatment of previously diagnosed or specific treatment or medications for hypertension.
5. Fasting plasma glucose (FPG) ≥ 100 mg/dL (5.6 mmol/L) or previously diagnosed or specific treatment or medications for type 2 diabetes.

Since there were some missing data in some of these variables, the dichotomous variable of MetS was created based on available data for each participant. For instance, if a male participant with 110 cm waist circumference, 150 mg/dL FPG, and 160 mg/dL triglycerides where other two variables of HDL blood pressure were missing were considered a MetS case regardless of the status of two other variables.

### 2.3 Measurements

All anthropometric data of ACSA were measured and recorded by trained staff. To measure waist circumference, a tape ruler was used from the lower costal margin to the iliac crest. All subjects fasted for at least 12 h before laboratory tests, including fasting blood sugar, HDL cholesterol, and triglycerides. Standard laboratory methods were used to conduct all blood tests. The blood pressure was measured three times from both arms each time (after 10–15 min rest), and finally, the mean blood pressure of the left hand and, in case of inability to measure through the left hand, the right hand's blood pressure was considered systolic and diastolic blood pressure.

All NCD data were collected through self‐report based on previous physicians' diagnoses. Physical activity was scored based on the Physical Activity Scale for the Elderly (PASE) [18]. The sleep quality was also assessed using Pittsburgh Sleep Quality (PSQI). Sleeping problem is determined by a score above five on this 19‐item tool with seven subclasses. Cronbach's alpha calculated the reliability of the questionnaire as 0.83 [19].

### 2.4 Statistical Analysis

The mean and standard deviation were used to describe quantitative and qualitative variables, percentages, and numbers. Two‐sided T‐test and chi‐square test were used for testing quantitative and qualitative variables in Table 1. First, a univariable model (α = 0.2) was used to test the adjusted associations of independent variables, followed by a multivariable model (α = 0.05). In addition to the overall *p* value of the final model, adjusted beta coefficients, statistical significance (*p* value), and confidence intervals of the beta were provided. The significance level (alpha) was considered as 0.05 in all tests. The analyses were conducted using Stata Corp Stata version 14.1.

**Table 1:** Baseline characteristics of participants (*n* = 5944).

Variables	Total *n* (%)	Metabolic syndrome, *n* (%)		
		No (*n* = 1973)	Yes (*n* = 3971)	*p* value
Age, mean (SD)	62.49 (7.98)	61.02 (7.87)	63.21 (7.92)	< 0.001
**Age group**				
Middle‐aged (50–60)	2464 (41.5)	992 (50.3)	1472 (37.1)	< 0.001
Elderly (> 60)	3480 (58.5)	981 (49.7)	2499 (62.9)	
**Gender**				
Male	2944 (49.5)	1142 (57.9)	1802 (45.4)	< 0.001
Female	3000 (50.5)	831 (42.1)	2169 (54.6)	
**Marital status**				
Single^a^	522 (8.8)	114 (5.8)	408 (10.3)	< 0.001
Married	5422 (91.2)	1859 (94.2)	3563 (89.7)	
**Education**				
Illiterate	772 (13.0)	207 (10.5)	565 (14.3)	
Elementary school	2801 (47.2)	873 (44.3)	1928 (48.7)	
Middle school	886 (14.9)	332 (16.8)	554 (14.0)	
High school	739 (12.5)	265 (13.4)	474 (12.0)	
College	735 (12.4)	295 (15.0)	440 (11.1)	< 0.001
**Smoking**				
Never	4299 (75.2)	1388 (72.0)	2911 (76.9)	< 0.001
Former	621 (10.9)	195 (10.1)	426 (11.3)	
Current	795 (13.9)	349 (17.9)	449 (11.9)	
**Family History**				
Type 2 diabetes	3539 (62.0)	1057 (54.9)	2482 (65.6)	< 0.001
Hypertension	3729 (65.4)	1152 (59.8)	2577 (68.2)	
Physical activity,^b^ mean (SD)	139.01 (86.64)	157.31 (93.88)	129.92 (81.30)	< 0.001
BMI, mean (SD)	28.54 (4.88)	26.32 (4.65)	29.70 (4.59)	< 0.001
Type 2 diabetes	2340 (39.4)	260 (13.2)	2080 (52.4)	< 0.001
Fatty liver	734 (13.1)	140 (7.4)	594 (16.0)	< 0.001
Thyroid disorders	718 (12.8)	189 (10.0)	529 (14.2)	< 0.001
Osteoporosis	934 (16.3)	225 (11.7)	709 (18.7)	< 0.001
Cardiovascular disorders	1080 (18.3)	107 (5.5)	973 (24.8)	< 0.001
**Sleep quality (PSQI)**				
Good	1578 (28.0)	631 (33.2)	947 (25.4)	< 0.001
Poor	4049 (72.0)	1271 (66.8)	2778 (74.6)	

^a^ Include never married, divorced, and widowed.

^b^ PASE score.

## 3 Results

A total of 5944 older adult participants entered this study analysis. The mean age was 62.5 ± 8.0, and 50.5% were female. Most of the participants (91.2%) were married, literate (87.0%), and never smoked (75.2%). It should be noted that less than 1% of female participants had ever smoked in their life. The mean physical activity score (PASE) was 139.0 ± 86.4, and the mean BMI was 28.5 ± 4.9. More than half of the study population reported a family history of diabetes (62%) and hypertension (65.4%). Furthermore, based on the self‐reported data, the prevalence of diabetes, fatty liver, thyroid disorders, osteoporosis, and cardiovascular disorders were 39.4%, 13.1%, 12.8%, 16.3%, and 18.3%, respectively. Also, 76% of participants reported having poor sleep quality based on PSQI (Table 1).

**Figure 1:**
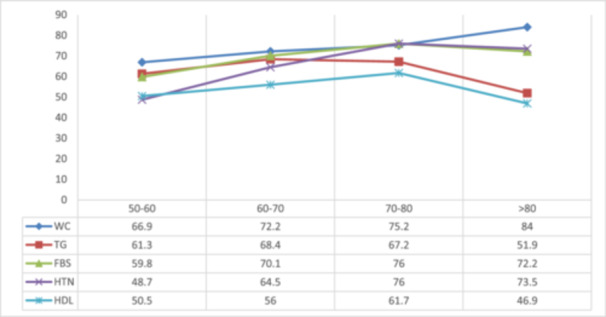
The prevalence (%) of MetS components in different age groups.

The prevalence of MetS was 66.81% (95% CI: 65.59–68.00) in this study. Based on Table 2, the most prevalent MetS components among study participants were triglyceride, waist circumference, and FPG, respectively. However, the HDL cholesterol component was the least prevalent one. People with MetS had 4.0 ± 0.8 of components, and on the other hand, participants without MetS had 1.3 ± 0.7 components. HDL and TG were less prevalent in older ages, while WC, HTN, and FBS were more prevalent (Figure 1).

**Table 2:** Prevalence of metabolic syndrome components (*n* = 5944).

Components	Metabolic syndrome, *n*(%)	
	No (*n *= 1973)	Yes (*n* = 3971)
**Waist circumference** > = 102/88 cm	862 (43.7)	3329 (83.8)
**Triglycerides** > = 150 mg/dL	430 (21.8)	3406 (85.8)
**HDL cholesterol** < 40/50 mg/dL	230 (11.7)	2980 (75.0)
**Hypertension** ≥ 85/130	489 (24.8)	3022 (76.1)
**Fasting plasma glucose** ≥ 100 mg/dL	676 (34.3)	3260 (82.1)
**Total met criteria,** mean (SD)	1.3 (0.7)	4.0 (0.8)

**Figure 2:**
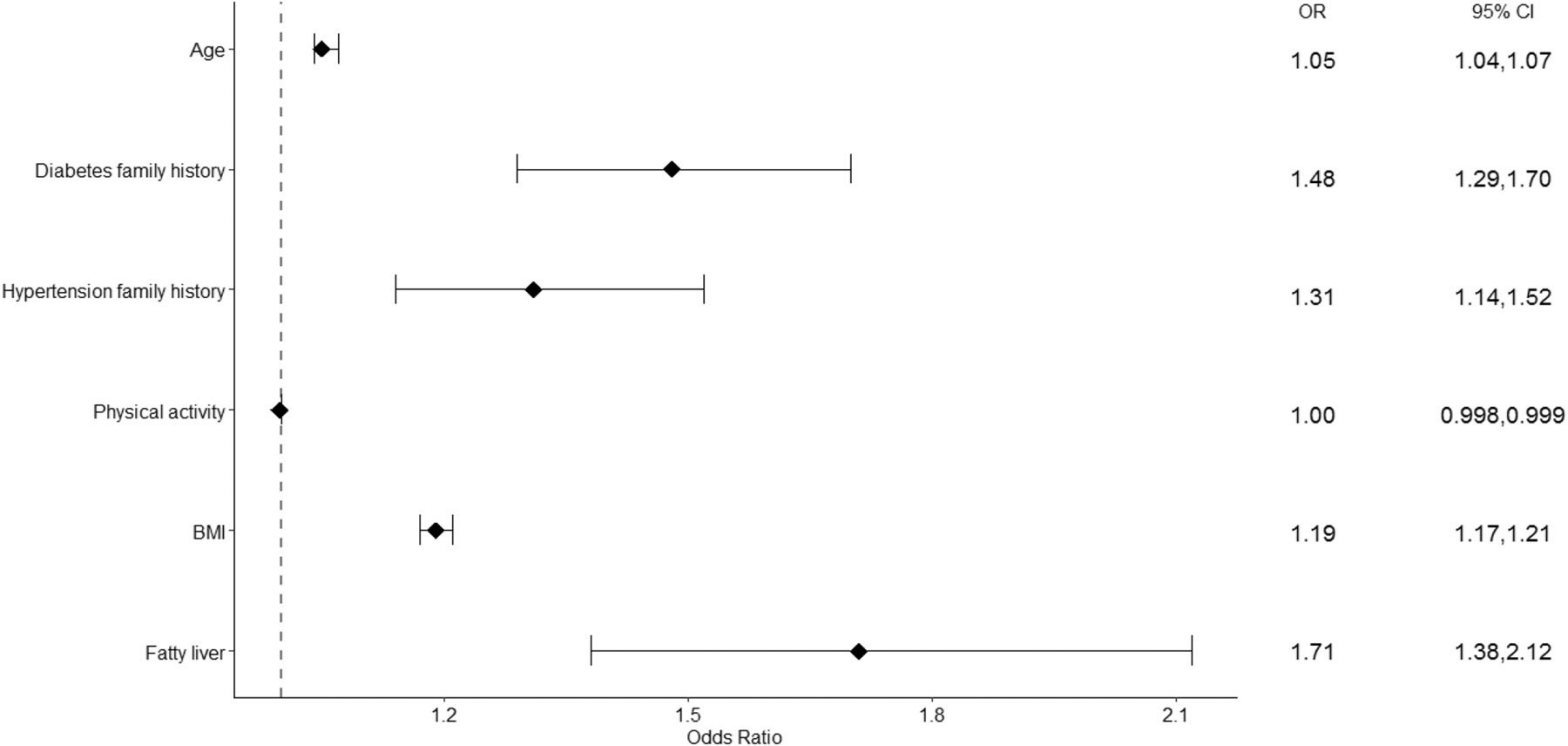
Forest plot of factors associated with MetS.

Table 3 and Figure 2 show the results of logistic regression for factors affecting MetS. Based on the univariable model (crude OR) and on a 0.2 level of significance, all variables were eligible to be entered into the multivariable model (α > 0.2).

**Table 3:** Logistic regression results for factors associated with MetS (*n* = 5944).

Covariates (reference level)	Univariable (α < 0.2)			Multivariable (α < 0.05)		
	crude OR	95% CI	*p* value	adjusted OR	95% CI	*p* value
**Age**	1.04	1.03–1.04	< 0.001	1.05	1.04–1.07	< 0.001
**Sex** (female)	0.60	0.54–0.67	< 0.001	—	—	—
**Marital status** (single)	0.54	0.43–0.66	< 0.001	—	—	—
**Education** (Illiterate)						
Elementary	0.81	0.67–0.97	0.02			
Middle school	0.61	0.50–0.75	< 0.001	—	—	—
High school	0.65	0.53–0.82	< 0.001			
College	0.55	0.44–0.67	< 0.001			
**Smoking** (never)						
Former	1.04	0.87–1.24	0.66			
Current	0.62	0.53–0.72	< 0.001	—	—	—
**Diabetes family history**	1.57	1.40–1.75	< 0.001	1.48	1.29–1.70	< 0.001
**Hypertension family history**	1.44	1.28–1.61	< 0.001	1.31	1.14–1.52	< 0.001
**Physical activity (PASE)**	0.996	0.996–0.997	< 0.001	0.998	0.998–0.999	< 0.001
**BMI**	1.19	1.17–1.21	< 0.001	1.19	1.17–1.21	< 0.001
**Sleep Quality (PSQI)** (good)	1.46	1.29–1.64	< 0.001	—	—	—
**Fatty liver**	2.83	1.96–2.89	< 0.001	1.71	1.38–2.12	< 0.001
**Thyroid disorders**	1.50	1.25–1.78	< 0.001	**—**	—	—

Based on the multivariable regression, six variables of age, family history of diabetes and hypertension, physical activity, BMI, and fatty liver were associated with MetS. Each year increase in age increases the odds of having MetS by 5%. Family history of diabetes and hypertension increase the odds of MetS by 48% and 31%, respectively. Higher level/score of physical activity is associated with less odds of having MetS. Fatty liver increases the odds of MetS by 1.71 folds. BMI also increases the odds of MetS by 19% for each unit of increase.

## 4 Discussion

In this study, based on the ATP III definition, we aimed to estimate the prevalence of Metabolic Syndrome (MetS) in older adults of Ardakan, Yazd, Iran. We also evaluated some of the essential factors associated with MetS. The main finding of this study was that the prevalence of MetS in older adults of Ardakan is 66.8%. It should be noted that since this study is performed based on a cross‐sectional phase of a cohort study, all observed associations could be biased (temporal bias) or misleading and, therefore, should be interpreted with caution to avoid any false discussion or conclusion.

Since the prevalence of diabetes in Yazd province is shown to be highest in Iran [20], and also since participants of the ACSA cohort and this study were over 50‐year‐old adults and the nature of this study and its correlation with age, we expected to observe the high prevalence of MetS in participants of this study. Hence, as expected, the prevalence of MetS was estimated as high as 66.8% among old adults of Ardakan. In a previous meta‐analysis study in Iran, the overall prevalence of MetS reported as 30.4%, which differs from the lowest in Sistan and Baluchestan Province (18.3%) to the highest in Bushehr (57.8%) [21]. In another meta‐analysis on the prevalence of MetS in Middle East countries, MetS was estimated to be 6%–42% in Iran [7]. The results of this study are in concordance with other studies [9, 22].

As mentioned, this syndrome is known as one of the late‐life diseases which is expected to occur mostly during the second 50 years of an individual's lifespan. So, we expect that the prevalence of this syndrome be higher in older adults, which was shown in this study. However, as Figure 1 shows, the components of this syndrome could be different in each age group. A notable finding that was mentioned and found in other studies [23, –, 25] as well and which could be used in clinical and prevention decision‐making settings.

Although this study is focused on reporting the prevalence of MetS in older Iranian adults, a series of analytic tests were performed to explore further and add to the current literature regarding factors associated with this syndrome. Results of our final model showed that older age, having a family history of hypertension and diabetes, low physical activity, having a higher BMI and fatty liver increase the odds of having MetS. However, as mentioned above, this result could be under the temporal bias [26] effect, but since these results have also been shown in recent studies, they can be relied on and accepted.

The findings of this study show the significant association between MetS and a family history of diabetes and hypertension, emphasizing the combined impact of genetic and environmental factors in its development. Individuals with a first‐degree relative affected by type 2 diabetes or hypertension exhibit a higher likelihood of developing MetS components, such as obesity, dyslipidemia, and insulin resistance [27, 28]. Shared genetic factors, such as variations in genes influencing insulin sensitivity, alongside common environmental exposures and lifestyle patterns, contribute to this heightened susceptibility [29, 30]. Psychosocial factors, including stress and health‐related behaviors shaped by family health patterns, further mediate this relationship [31].

The association between fatty liver and MetS, shown in this study, is also reported in previous studies [32, 33]. This disease is considered a cause and a consequence of MetS [34]. Since the fatty liver is highly associated with obesity [35] and also regarding the correlation of BMI and MetS, this association is reasonable. In our study, the prevalence of fatty liver and above‐30 BMI cases was higher in females; as a result, the prevalence of MetS was higher in females.

This study was part of the Ardakan Cohort Study on Aging (ACSA) of more than 5944 elderly individuals. Accordingly, the findings could represent the elderly population of Iran and other countries with similar demographic characteristics. Due to the large sample size and random sampling, this study can be referred to and used with a high degree of representativeness. Since this is the first phase of a cohort study, it is impossible to interpret causality in this study because it is cross‐sectional. Other factors may also influence the observed association, so the results of this study are only a tiny part of a more extensive network of associations in the context of MetS.

## 5 Conclusion

This study showed that the prevalence of metabolic syndrome was high in the elderly population of Iran. Also, the most prevalent MetS components were triglyceride, waist circumference, and FPG and HDL was the least prevalent one. Additionally, the prevalence of MetS components was different in age groups, which should be noted by policy and decision‐makers. Furthermore, this study showed that older age, having a family history of hypertension and diabetes, low physical activity, having a higher BMI and fatty liver are more susceptible to have MetS. As was shown regarding the high prevalence of MetS in older Iranian adults, tailored interventions and controlling measures seem critical and urgent to lower this prevalence. Also, noting that the most prevalent components of MetS in this population, weight control, healthy nutritional behaviors, and hyperlipidemia prevention plans, should be at the center of decision‐makings.

## Author Contributions

**Elham Hooshmand:** conceptualization, methodology, validation, software, data curation, supervision, resources, project administration, formal analysis, visualization, writing – review and editing, writing – original draft, funding acquisition, investigation. **Isa Akbarzade:** conceptualization, methodology, validation, investigation, funding acquisition, writing – original draft, writing – review and editing, data curation, software, resources, project administration. **Delaram Delbari:** conceptualization, validation, writing – original draft, writing – review and editing, formal analysis, supervision, resources, investigation, methodology. **Mahtab Niroomand:** conceptualization, investigation, funding acquisition, writing – review and editing, writing – original draft, methodology, validation, data curation. **Fatemeh Ghavidel:** conceptualization, data curation, software, methodology, validation, formal analysis, writing – original draft, writing – review and editing, investigation. **Mohammad Saatchi:** writing – original draft, writing – review and editing, project administration, resources, data curation, supervision, software, formal analysis, visualization, validation, methodology, conceptualization, investigation, funding acquisition.

## Ethics Statement

All authors have read and approved the final version of the manuscript. Dr. Mohammad Saatchi had full access to all of the data in this study and took complete responsibility for the integrity of the data and the accuracy of the data analysis. All authors are primarily involved in education or research and are not directly supported by the government.

## Conflicts of Interest

The authors declare no conflicts of interest.

### 1 Transparency Statement

The lead author Mohammad Saatchi affirms that this manuscript is an honest, accurate, and transparent account of the study being reported; that no important aspects of the study have been omitted; and that any discrepancies from the study as planned (and, if relevant, registered) have been explained.

## Data Availability

The data that support the findings of this study are available on request from the corresponding author. The data are not publicly available due to privacy or ethical restrictions.
